# Oxaliplatin long-circulating liposomes improved therapeutic index of colorectal carcinoma

**DOI:** 10.1186/1472-6750-11-21

**Published:** 2011-03-15

**Authors:** Chuang Yang, Hai Z Liu, Zhong X Fu, Wei D Lu

**Affiliations:** 1 Department of Gastrointestinal Surgery, First Affiliated Hospital, Chongqing Medical University, Chongqing 400016, Chongqing, China; 2 Department of Hepatobiliary Surgery, Mianyang Third People's Hospital, Mianyang 621000, Sichuan Province, China; 3 Departments of Gynecology and Obstetrics, Second Affiliated Hospital, Chongqing Medical University, Chongqing 400016, Chongqing, China

## Abstract

**Background:**

Cytotoxic drugs are non-selective between normal and pathological tissue, and this poses a challenge regarding the strategy for treatment of tumors. To achieve sufficient antitumor activity for colorectal carcinoma, optimization of the therapeutic regimen is of great importance. We investigated the ability of oxaliplatin long-circulating liposomes (PEG-liposomal L-oHP) to provide an improved therapeutic index of colorectal carcinoma.

**Results:**

We determined that PEG- liposomes conjugated with cells at 2 h, with a mean fluorescence intensity that was enhanced upon extended induction time. The PEG-liposomal L-oHP induced a significant apoptotic response as compared with free L-oHP, 23.21% ± 3.38% vs. 16.85% ± 0.98%, respectively. Fluorescence imaging with In-Vivo Imaging demonstrated that PEG- liposomes specifically targeted tumour tissue. After intravenous injections of PEG-liposomal L-oHP or free L-oHP, the tumour volume suppression ratio was 26.08% ± 12.43% and 18.19% ± 7.09%, respectively, the percentage increased life span (ILS%) was 45.36% and 76.19%, respectively, and Bcl-2, Bax mRNA and protein expression in tumour tissue was 0.27-fold vs. 0.88-fold and 1.32-fold vs. 1.61-fold compared with free L-oHP, respectively.

**Conclusion:**

The PEG-liposomal L-oHP exhibited a tendency to target tumour tissue and demonstrated a significantly greater impact on apoptosis compared to free oxaliplatin.

## Background

Colorectal carcinoma (CRC) is the third most common form of cancer in the world, and the rectum exhibits common internal malignancies [1]. Oxaliplatin (L-oHP) is a third generation platinum antitumor compound. Clinically, it is now approved as first-line chemotherapy in combination with other antitumor drugs for the treatment of advanced colorectal cancer [2,3]. It contains a bulky carrier ligand within its structure, and forms DNA adducts that more effectively inhibit DNA synthesis; however, these adducts are generally considered to be more cytotoxic than those of either cisplatin or carboplatin [4,5]. Cytotoxic drugs exhibit obvious toxicity on the human body, affecting neurotoxicity, gastrointestinal reaction, and cardiotoxicity, etc. [6]; moreover, the non-selective nature of cytotoxic drugs regarding normal and pathological tissue poses a challenge for the treatment of tumors. Conventional chemotherapy is not as effective in colorectal cancer as it is in other cancers since the drug does not reach the target site in an effective concentration [7,8]. Thus, effective treatment demands an increased dose, which may lead to negative side effects. If drugs were targeted to the tumor cells, these limitations would be overcome, and this in turn would be advantageous for the cancer treatment.

Liposomes are small, spherically shape vesicles that can be produced from cholesterols, non-toxic surfactants, sphingolipids, glycolipids, long chain fatty acids and even membrane proteins. Liposomes were among the first nanomolecular drug delivery systems to demonstrate the increased delivery of small molecular weight anticancer drugs to solid tumors by altering the biodistribution of associated drugs [9,10]. It has been previously reported that liposomes attach to cellular membranes and appear to fuse with them, thus releasing their contents into the cells [11]. Alternatively, liposomes are taken up by the cell, their phospholipids are incorporated into the cell membrane, and the drug trapped inside is then released [12]. Common liposomes, though, were in the body for only a short duration, and many were phagocytized by the reticuloendothelial system (RES). However, the 1,2-distearoyl-sn-glycero-3-phosphoethanolamine-N-[maleimide(polyethylene glycol)-2000] (DSPE-PEG2000) modification to the surface of a liposome potentially prevents interactions *in-vivo*, thus extending the circulation lifetime of the liposome [13-15]. In tumor tissue, because tumor cells grow so quickly, newly formed tumor vessels are comprised of poorly-aligned and defective endothelial cells with wide fenestrations that lack a smooth muscle layer and innervation with the wider lumen. Furthermore, tumor tissues usually lack effective lymphatic drainage [16]. Tumor microvessel permeability is 400~600 nm with permeability for macromolecules having a molecular weight of 2.5 × 10^4^~16 × 10^4 ^Da [17]. These factors lead to abnormal molecular and fluid transport dynamics. Therefore, enhancement of the extravasation of certain sizes of molecules, such as macromolecular drugs or liposomes, leads to a much greater accumulation in tumour tissue versus normal tissue. Due to the tumor selective enhanced permeability and retention effect (EPR), this results in extensive extravasation of the liposomes [16,18]. In solid tumours, the EPR effect is a universal phenomenon in which liposomes are passively targeted to tumour tissue, ultimately leading to enhanced accumulation of the liposomes in the tumor interstitium [19].

Recently, FAD of USA approved a few liposomal products, such as Evacet, AM Bison, and doxorubicin in a long-circulating PEG-coated liposome. There have been initial reports indicating that the use of individual functionalities has been demonstrated to be associated with highly positive clinical outcomes [20-22]. However, there is currently no commercially available PEG-liposomal L-oHP product, and studies are still in the experimental stage. There are few reports published regarding PEG-liposomal L-oHP treatment of colorectal cancer. Here, we investigated the therapeutic tumour targeting activity of PEG-liposomal L-oHP *in-vitro *in SW480 cells and *in-vivo *in a nude mice solid tumour model.

## Results

### Characteristics of long-circulating liposomes (PEG-liposomes) and cellular uptake

**Figure 1:**
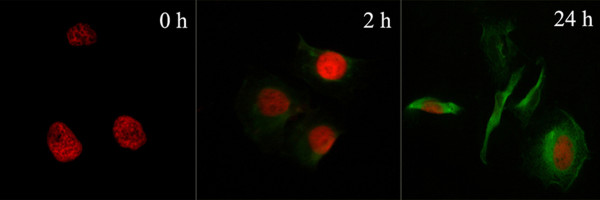
**Cellular uptake of liposomes**. The liposomes conjugated with cells after 2 h, and cellular uptake of liposomes increased over time. Cells were incubated with PI, which stained nuclei red, and Dio-labelled liposomes, which were green in colour. A considerable number of liposomes aggregated within cells at 24 h (× 400).

We selected an increased particle size of PEG-liposomes through a series of filtration steps using a polycarbonate membrane filter at a pore size of 100 nm. PEG-liposomes with a particle size of 151.56 ± 15.57 nm and zeta-potential of -23.68 ± 2.35 mv were obtained, as determined by laser grain size analysis. The entrapment efficiency of the liposomes was (42.96 ± 6.45)% as determined by HPLC. These values are higher than those reported recently by another group. Flow cytometry demonstrated that after incubation in medium containing Dio-labeled liposomes for 2 h, the PEG-liposomes conjugated with cells, and exhibited an enhanced mean fluorescence intensity upon extended induction time; the mean fluorescence intensity at 24 h was 3.28-fold greater than the intensity measured at 2 h. The immunofluorescence assay revealed considerable aggregation of liposomes within cells at 24 h (Figure 1).

### In-vitro drug release and cell viability assay

We used the dialysis method to evaluate L-oHP release from encapsulated PGE-liposomes *in-vitro*, and the drug concentration was then analyzed by HPLC. The cumulative percentage release demonstrated that the amount of drug released from PEG-liposomes was gradually increased over time, and after 120 h there was an increase of over 89%. The free drug exhibited the highest level (94%) at 2 h, confirming the fact that PEG-liposomes act as a barrier against diffusion of hydrophilic drugs.

The viability of cells was analyzed by the MTT colorimetric assay after treatment with empty PEG-liposomes (2.6 μmol/ml), free L-oHP (28 μg/ml) and PEG-liposomal L-oHP (containing L-oHP 28 μg/ml), respectively. Cell viability was decreased with the length of exposure, with a maximum reduction occurring at 12 h. The empty PEG-liposomes exhibited significantly less cytotoxicity.

### Analysis of apoptosis

**Figure 2:**
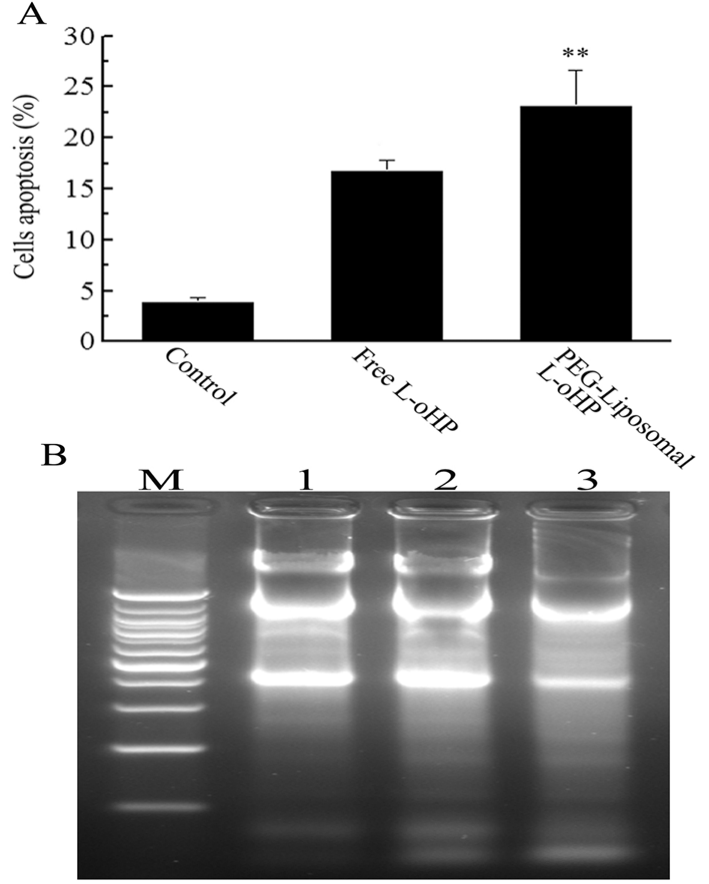
**Apoptosis of SW480 cells**. SW480 cells treated with free L-oHP (28 μg/ml) or PEG-Liposomal L-oHP (containing L-oHP 28 μg/ml), PEG-Liposomal L-oHP induced marked apoptosis compared with free L-oHP (P < 0.01). A, flow cytometery; B, DNA fragmentation. M: marker (4000 bp). 1: Control. 2: Free L-oHP. 3: PEG-liposomal L-oHP.

Upon exposure of SW480 cells to free L-oHP or PEG-liposomal L-oHP, cellular apoptosis was assessed by flow cytometry, which demonstrated that PEG-liposomal L-oHP induced SW480 apoptotic incidence of (23.21 ± 3.38)% (Figure 2A). The gel electrophoretic analysis of internucleosomal DNA fragmentation demonstrated the presence of primarily high molecular weight DNA as seen with the untreated control. A DNA ladder pattern, the typical feature of apoptosis, was distinctly observed (Figure 2B).

### Tumour tissue and Dio-labeled liposomes

**Figure 3:**
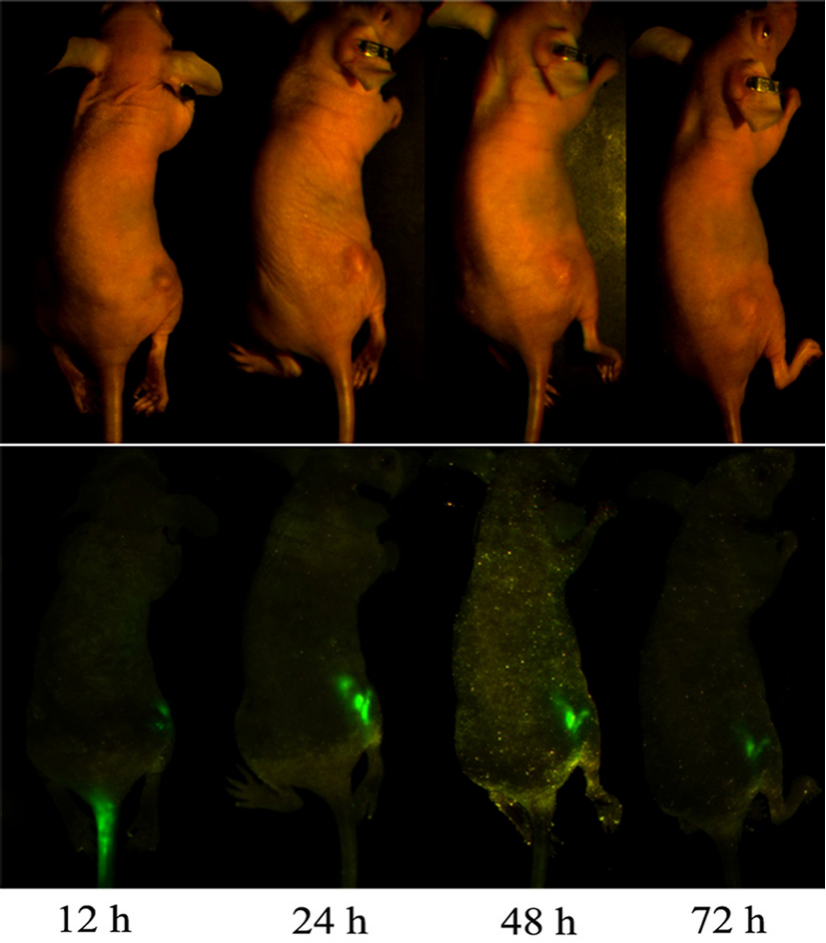
**Control mice intravenously injected with Dio-labeled liposomes at 12 h, 24 h, 48 h, and 72 h**. On day 10 after tumor inoculation mice received an intravenous injection of fluorescently (Dio) labeled empty PEG-liposomes (no drug) at a dose of 0.5 μmol lipid/g. At 12, 24, 48, and 72 h post-injection, *in-vivo *images were created.

Dio-labeled liposomes were intravenously injected via the tail vein (after injection, all mice survived), and then visualized in the tumour tissue by an *in-vivo *imaging system. After 12 h, 24 h, 48 h, and 72 h, typical representative images were captured (Figure 3). The fluorescence intensity distribution of tumour tissue in the animals was indicated by green fluorescence. The fluorescence intensity was maintained at a high level for an extended period of 24 h. However, immediately following intravenous injections, little fluorescence was observed, excluding part of the tail. The fluorescence was observed through 72 h, indicating that PEG-liposomes may continue accumulation.

### In-vivo antitumor effect of PEG-liposomal L-oHP

**Figure 4:**
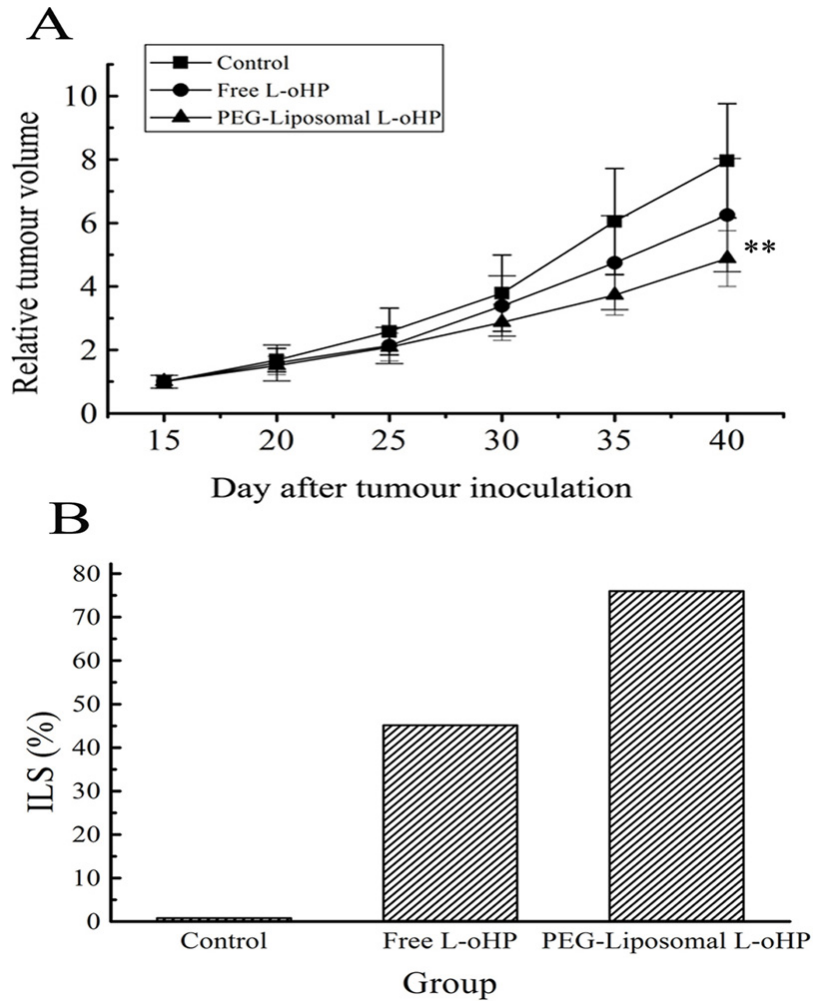
**Tumor growth suppression and survival time**. On day 12 after tumour inoculation, mice were treated ith free L-oHP (5 μg/g), PEG-liposomal L-oHP (containing L-oHP 5 μg/g), or 5% dextrose solution. Antitumor activity as assessed by tumour size and survival of tumour-bearing mice. A, Relative tumor volume (RTV), data represent mean ± SD (n = 6). ** P < 0.01 compared with the other group. B, The percentage increase in life span (ILS%).

Rapid tumour growth was observed in the mouse control group; however, significant tumor growth suppression was demonstrated in mice treated with PEG-liposomal L-oHP (Figure 4A). The tumour suppression was (26.08 ± 12.43)%, and PEG-liposomal L-oHP demonstrated the strongest effect on the survival time - all of the mice treated with PEG-liposomal L-oHP became long-term survivors (Figure 4B) (p < 0.01). Throughout the therapeutic experiment, a noticeable cachexia condition was observed in the control group, and although no bodyweight loss was observed in any of the treated groups, weight loss was significant in the control group (data not show). These results suggest that treatment with PEG-liposomal L-oHP improves the median survival time (MST) of tumor-bearing mice without causing remarkable toxicity.

### Bcl-2, Bax mRNA and protein expression in tumour tissue

**Figure 5:**
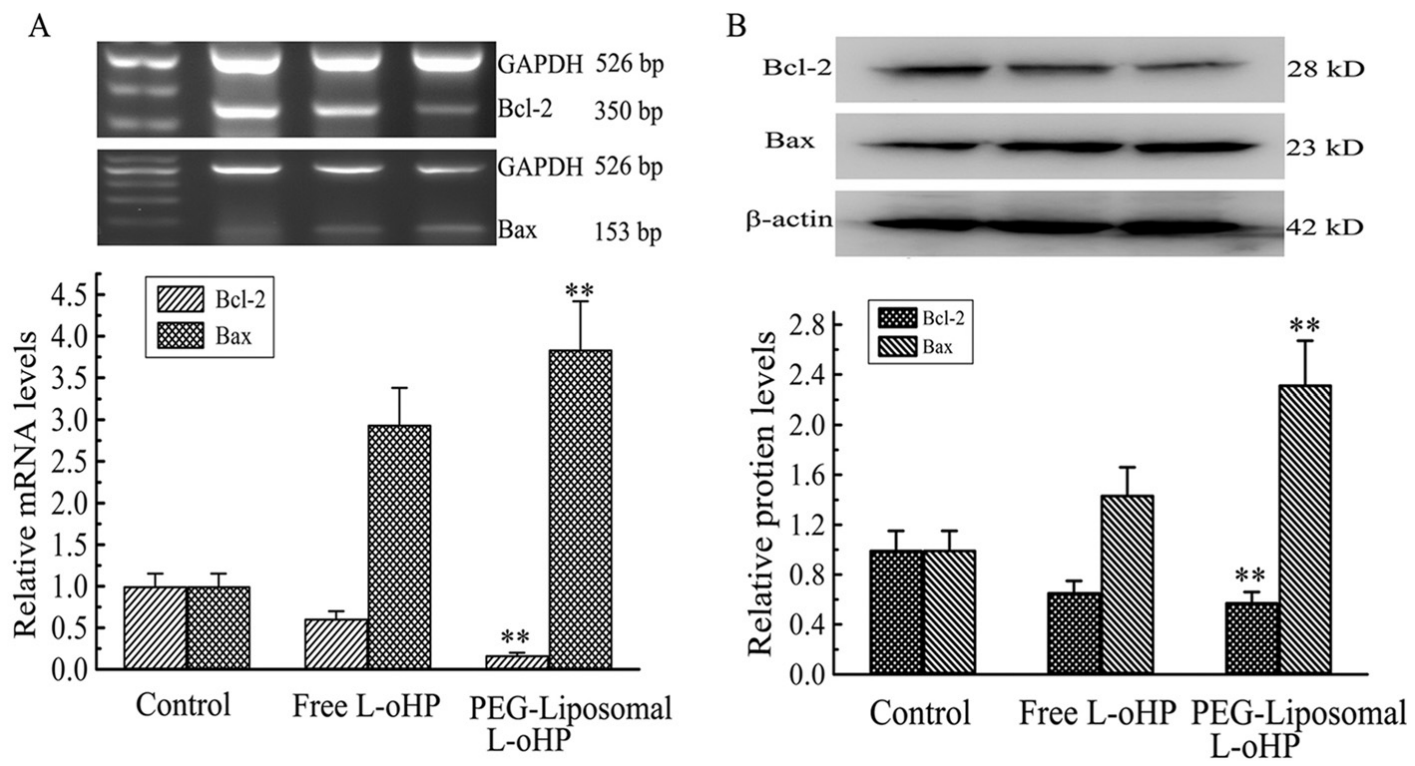
**The mRNA and protein expression of Bcl-2 and Bax in tumour tissue**. On day 15 after treatment, the total mRNA and protein was extracted from tumour tissue. A, The mRNA expression. Agarose gel electrophoresis result shown in which expression of Bcl-2 is down-regulated and expression of Bax is up-regulated; B, The protein expression. Cell lysates were separated on 12% SDS-PAGE gels, Bcl-2 was down-regulated, whereas Bax was up-regulated. Data are expressed as mean ± SD (n = 3). ** P < 0.01 compared with the other group.

To elucidate whether the growth inhibitory effect of PEG-liposomal L-oHP was attributable to the induction of apoptosis, Bcl-2 and Bax were analyzed by RT-PCR or Western blot in tumour tissue. On day 15 after treatment, tumours were resected and total RNA and protein were extracted from the tumour tissue. Our experiments demonstrated that mRNA expression levels of Bcl-2 were remarkably decreased in the PEG-liposomal L-oHP group; 0.27-fold compared with free L-oHP, whereas, Bax mRNA increased 1.32-fold compared with free L-oHP (Figure 5A). Protein expression tendency of Bcl-2 and Bax were 0.88-fold and 1.61-fold in comparison, respectively (Figure 5B). These results indicated that apoptosis was strongly induced by PEG-liopsomal L-oHP.

## Discussion

The non-selectivity of cytotoxic drugs between normal tissue and the pathological site poses a tumor treatment strategy challenge. To obtain increased therapeutic efficacy, a drug carrier must achieve increased delivery of the drug to the tumor tissue, while also allowing for enhanced interaction of the drug with, and subsequent internalization by, tumor cells. Liposomes, as carriers of chemotherapeutic agents, are able to change the distribution of these agents within the body and decrease their toxicity [23,24]. Therefore, drug-loaded liposomes offer a new approach for the treatment of colorectal cancer.

Polyethylene glycol (PEG)-coated liposomes (PEG-liposomes), are stable and not easy to be taken up by cells of the reticuloendothelial system (RES), and exhibit reduced drug leakage compared with conventional liposomes [25]. It has been previously reported that PEG modification of liposomes increases their affinity to cancer cells and increases the cellular uptake of drugs [26-28]. The toxicity of PEG-liposomes for cells should be taken into consideration, and some previous reports have indicated that the toxicity is indeed lower [29,30]. In our experiment, the empty PEG-liposomes *in-vitro *exhibited significantly less cytotoxicity for SW480 cells. The tumour cells took up large numbers of PEG- liposomes, which is in concordance with reports by other groups [31]. However, PEG-liposomes containing a drug increase toxicity. Our MTT assays showed that PEG-liposomal L-oHP (containing L-oHP 28 μg/ml) had significantly greater cytotoxic effects than free oxaliplatin (28 μg/ml). When we assessed their effects on apoptosis, as determined by flow cytometry and the DNA Ladder method, we observed that, at an identical dose, PEG-liposomal L-oHP demonstrated a significantly greater effect on apoptosis than did free L-oHP. PEG-liposomes exhibited tumour-targeted delivery in these cells.

Previous studies have demonstrated that PEG-modified liposomes act primarily through vesicular organelles, and are preferentially taken up by angiogenic tumour endothelium [32]. To obtain sufficient antitumor activity with liposomal anticancer drugs, optimization of the therapeutic regimen is of great importance. In solid tumours (during the rapid growth of the tumour in particular), the permeability of the vasculature is generally increased compared to normal tissues [32,33]. Therefore, these may provide a channel allowing liposomes to more easily target tumour tissue. After receiving intravenous injections of Dio-labeled PEG-liposomes, mice were able to survive. Experiments presented in this study indicate that PEG-liposomes efficiently accumulate in tumor tissue (Figure 3), and maintain a high level over 24 h, which is in concordance with previous reports from other groups [31,34]. Furthermore, the fluorescence remained detectable even after 72 h. The plasma clearance of anionic molecules occurred more slowly than for cationic molecules [35]. Based upon evidence from the *in-vitro *cell experiments and the mouse tumour model, a higher concentration and longer blood residence time of liposomes would result in greater efficiency of extravasation per unit volume of convective transport [36,37], and this would explain the fact that liposomes remain in the tumor tissue.

Additionally, to investigate the treatment availability of PEG-liposomal L-oHP, Bcl-2 and Bax were evaluated. Bcl-2 and Bax are members of the Bcl-2 family, Bax is a proapoptotic protein that induces mitochondrial outer membrane permeabilization (MOMP), causing the release of caspase activating proteins. In contrast, Bcl-2 is an anti-apoptotic protein and guardian of the outer membrane and it preserves its integrity by opposing Bax; they are associated with apoptosis necrosis, and autophagy, and regulate all major types of cell death [38,39]. We used the level of genes and protein to indicate treatment results. After treatment with PEG-liposomal L-oHP, tumour cell predominance of apoptosis in tumor-bearing nude mice was induced, and Bcl-2 mRNA and protein expression were down-regulated, whereas Bax was up-regulated (Figure 5). This demonstrated that such liposomal L-oHP formulation exhibits potent *in-vivo *antitumor activity, presumably via a dual targeting approach against both tumour endothelial cells and tumour cells [40,41].

The PEG-liposomal L-oHP accumulated in the tumour tissue, following uptake by endothelial cells as well as tumor cells, and liposomes were then degraded, while intracellular drug delivery increased the concentration of drug within cells and slowed drug efflux [42-44]. These findings indicate that liposome encapsulation of chemotherapeutic drugs enhances their damaging effects on tumour cells. At present, FDA approved liposomal products (Evacet, doxorubicin liposomes, etc.) have the advantage of high encapsulation efficiency, rapid release rate, and so forth. As to our study, further research is needed in order to improve drug encapsulation efficiency and stability, as well as further studies involving dynamic research in a clinical setting.

## Conclusion

The experiments presented in this report indicate that PEG-liposomal L-oHP achieves a better therapeutic response than the equivalent dose of free L-oHP, and it indicates the potentially wide application for this type of drug target for tumors and other tissues, with the advantage of the ability to overcome some major limitations in conventional anticancer chemotherapy. This study may provide the rationale for the clinical application of CRC. Nevertheless, further studies are warranted to elucidate the underlying molecular mechanism.

## Methods

### Animals and tumor cell line

Female BALB/c nude mice, 3 weeks old, were obtained from Center of Laboratory Animals, Chongqing Medical University (Permit Number: SCXK(jing) 2009-0004). All animal experiments were evaluated and approved by the Animal and Ethics Review Committee. The human colorectal carcinoma cell line (SW480) was obtained from the Institute of Life Science of Chongqing Medical University, and it was maintained in RPMI 1640 (Sigma, St. Louis, MO) supplemented with 10% fetal bovine serum (FBS) (HyClone, Logan, UT) in a 5% CO_**2 **_incubator at 37°C.

### Preparation of liposomes

PEG-liposomes were prepared using lecithin (Sigama Co., US), cholesterol (Sigama Co., US), and DSPE-PEG2000 (Avanti Polar Lipids Inc. US) as previously described [31]. The molar ratio was 2.0:1.0:0.2. In the targeting experiments, 2 nmol/ml of the fluorescent lipid membrane marker, Dio (Vigorous Biotech Co. Ltd. China), was added to the lipid mixture. The liposomes were prepared using the reverse-phase evaporation method. Briefly, lipids (50 mmol) were dissolved in 15 ml of chloroform and then 5 ml of L-oHP solution (1 mg/ml) in 5% (w/v) dextrose was dropped into the lipid mixture to form W/O emulsion. For preparation of no drug-containing liposomes, 5% dextrose solution was added instead of L-oHP solution. The volume ratio of the aqueous to the organic phase was maintained at 1:3. The emulsion was sonicated for 10 min (40 W) and then the organic phase was removed to form the liposomes by evaporation in a rotary evaporator at 40°C under vacuum at 0.045 mPa for 2 h. The resulting liposomes were extruded through a polycarbonate membrane (Millipore, US. 100 nm pore size).

The grade size and zeta-potential were detected by Laser Particle Size Analyzer (Zetasizer, Malvern). Using a transmission electron microscope (Hitachi S-3000N, japan), the form feature of PEG-liposomes was determined. The free L-oHP was removed by ultrafiltration (MW 100 kDa, 12,000 r/min 20 min). The entrapment efficiency of the liposomes was determined by high-performance liquid chromatography (HPLC, SY-8100, Beijin, China). *In-vitro *drug release from PEG-liposomes was studied using a dialysis method as described by Zhang et al. [45].

### Cellular uptake of Dio-labeled PEG-liposomes

SW480 cells were seeded onto 6-well plates in 1 ml of RPMI 1640 medium containing 10% FBS and pre-incubated for 24 h. After removal of culture medium, 1 ml of fresh medium containing the Dio-labeled PEG-liposomes (2 μmol/ml) was added, followed by incubation at 37°C. At 0, 2, 4, 8, 12 and 24 h post-incubation, the cells were trypsinized, followed by two washes with cold phosphate buffered saline (PBS). The cells were re-suspended in 400 μl of PBS (1 × 10^6^/ml). The cellular uptake of Dio-labeled liposomes was quantified using a flow cytometer (FACS Aria, Becton, Dickinson and Company), equipped with an argon-ion laser and 488 nm band pass filters for emission measurements. Approximately 10,000 events were acquired per sample. Cells were also plated onto glass slides and incubated with Dio-labeled liposomes, and the cellular uptake of liposomes was determined by measuring fluorescence.

### Cytotoxicity assay

Cytotoxicity of L-oHP formulations was determined by the 3-(4,5-dimethylthiazol-2-yl)-2,5 diphenyl tetrazolium bromide (MTT) assay, as described previously [33]. Briefly, cells in the logarithmic growth phase (5 × 10^3 ^per well) were placed in wells of a 96-well plate and incubated for 24 h. The culture medium was replaced with fresh medium containing various concentrations of blank liposomes, free oxaliplatin, or PEG-liposomal L-oHP. After treatment, the culture medium was removed and the cells were incubated with MTT (final concentration 10%) for 4 h at 37°C. Then 150 μl DMSO was added to each well to dissolve formazan crystals. The absorbance of each well was read at 570 nm on a microplate reader, and used to determine IC50 values (IC50 values represent L-oHP concentrations that cause 50% cell death). The concentration of oxalipatin liposomes was expressed as 1/2 the IC50 of the working concentration of oxaliplatin.

### Detection of apoptosis by flow cytometry

SW480 cells cultured in 6-well plates were treated with free oxaliplatin (28 μg/ml) or PEG-liposomal L-oHP (containing L-oHP 28 μg/ml) for 12 h, along with a blank control with no drug treatment. The cells were trypsinized, followed by two washes with cold PBS, re-suspended in 400 μl of PBS (1 × 10^6^/ml), and incubated in the dark for 15 min following addition of Annexin V-FITC (5 μl). Cells were subsequently treated with PI (10 μl) and incubated in the dark for 5 min prior to detection by flow cytometry. Approximately 10,000 events were acquired per sample.

### DNA fragmentation analysis for detecting apoptosis

For DNA fragmentation assay, cells were treated as described above. Adherent and floating cells were recovered and DNA was isolated and evaluated for fragmentation as described previously [46]. DNA samples were applied on 1.5% agarose gel containing 1% GoldView™. The gel was examined and photographed using an ultraviolet gel documentation system (Bio-Rad, Hercules, CA, USA).

### Targeting of Dio-labeled liposomes in tumor-bearing nude mice

Female BALB/c nude mice were inoculated subcutaneously at the inguen region with SW480 Cells (2 × 10^7^/mouse) in a volume of 200 μl (PBS). On day 15 after tumor inoculation, the tumor volume reached approximately 100 mm^3^. Next, intravenous injections of Dio-labeled PEG-liposomes (0.5 μmol/g) were performed via the tail vein. At 12 h, 24 h, 48 h, and 72 h post-injection, nude mice were anesthetized with isoflurane, and fluorescence imaging was performed using the In-Vivo Imaging System (Mastro Ex, USA) which has an affiliated anesthesia device.

### Therapeutic efficacy of PEG-liposomal L-oHP in tumor-bearing nude mice

After successful subcutaneously inoculated tumor transplantation, the nude mice were randomly divided into three groups. Control (n = 6): Received intravenous injections of 5% dextrose solution; Free L-oHP (n = 6): Received intravenous injections of 5 μg L-oHP/g; PEG-liposomal L-oHP (n = 6): Received intravenous injections of PEG-liposomal L-oHP (containing L-oHP 5 μg/g). Treatments occurred once every four days, and the antitumor activity was evaluated in terms of both relative tumor volume (RTV) and the percentage of increased life span (ILS%). Tumor volume was calculated using the method described by Kim [47] and the ILS was calculated using the method described by Kviecinski [48]. The median survival time (MST) of each group was recorded.

### Reverse transcription-polymerase chain reaction (RT-PCR)

On day 15 after treatment, the nude mice were sacrificed by deep anesthesia, and the tumours were immediately placed in liquid nitrogen for further experiments. Total RNA was extracted using TRIZOL (Takara, Dalian, China). Reverse transcription was carried out in 10 μl of reaction mixture containing 1 μg of total RNA, 25 pmol of oligo-dT primer, 10 nmol of dNTP mixture, 20 units of RNase inhibitor, and 2.5 units of AMV reverse transcriptase (Takara, Dalian, China) at 37°C for 15 min, 85°C for 5 s. PCR amplification was performed in 25 μl PCR reaction mixture. PCR amplification was conducted to detect differences among the samples as follows: 4 min at 94°C for initial denaturation; 30 cycles × 30 s at 94°C, 30 s at 59°C, and 30 s at 72°C for Bcl-2, Bax; 30 cycles × 30 s at 94°C, 30 s at 60°C, and 30 s at 72°C for GAPDH. The following primer pairs were used: Bax (153 bp): 5'-GAT CGA GCA GGG CGA ATG GG-3' (ForwardPrimer); 5'-CAC GGC GGC AAT CAT CCT CT-3' (ReversePrimer); Bcl-2 (350 bp): 5'-CAG ATG GCA AAT GAC CAG CAGA-3' (ForwardPrimer), 5'-TGG CAG GAT AGC AGC ACA GGAT-3' (ReversePrimer); GAPDH (526 bp): 5'-AGG TCG GAG TCA ACG GAT TTG-3' (ForwardPrimer), 5'-GTG ATG GCA TGG ACT GTG GT-3' (ReversePrimer). For the analysis of PCR products, 6 μl of each PCR reaction was electrophoresed on 1.5% agarose gel containing 1% GoldView™. Band intensity was analyzed with Image system (NIH, USA) and GAPDH was used as an internal control to evaluate the relative expression of Bcl-2 and Bax.

### Western blot analysis

For isolation of total protein extract, tumour tissues were washed with ice-cold PBS and lysed in RIPA lysis buffer (50 mM Tris with pH 7.4, 150 mM NaCl, 1% Triton X-100, 1% sodium deoxycholate, 0.1% sodium dodecyl sulphate, and 0.05 mM EDTA) for 30 min on ice, and then the cell lysate was centrifuged (12,000 revs/min, at 4°C) for 10 min. The supernatant was collected and protein content of the extracted samples was measured using the Bradford protein assay kit (BestBo-BeiBo, Beijing, China). All samples were kept at -80°C for further experiments.

Levels of target proteins including Bcl-2, Bax (Santa Cruz Biotechnology, Inc. 1:200), and β-actin (Bioscience Company of America, 1:500) were determined by Western blot analysis using their respective antibodies. Briefly, total cell lysate was boiled in 5 × loading buffer (125 mM Tris-HCl, pH 6.8, 10% SDS, 8% dithiothreitol, 50% glycerol, and 0.5% bromchlorphenol blue) for 10 min. Equal amounts of proteins (50 μg) were subjected to 12% SDS-polyacrylamide gel electrophoresis and transferred to polyvinylidene fluoride membranes (PVDF). The membranes were blocked with 5% skim milk in PBS with 0.1% Tween 20 (PBST) for 1 h, and incubated with primary antibodies overnight at 4°C. Antibodies were detected by means of HRP-conjugated secondary antibody (Bioscience Company of America, 1:2000) for 1 h at room temperature. Immunoreactive bands were visualized using Immobilon™ Western Chemiluminescent HRP Substrate (Millipore, USA), and densitometric analysis was performed using the PDI Imageware System (Bio-Rad, Hercules, CA, USA).

## Abbreviations

CRC: Colorectal carcinoma; SDS-PAGE: Sodium Dodecyl Sulfate-Polyacrylamide Gel Electrophoresis; L-oHP: Oxaliplatin; DSPE-PEG2000: 1,2-distearoyl-sn-glycero-3-phosphoethanolamine- N-[maleimide(polyethylene glycol)-2000]; MTT: 3-(4,5)-dimethylthiahiazo (-z-y1)-3,5-di- Phenytetrazoliumromide; HPLC: High-performance liqued chromatography; DAB: 3,3'-diaminobenzidine; Dio: DIOC_18_(3), 3,3' dioctadecyloxacarbocyanine perchlorate; RES: Reticuloendothelial system EPR: Enhanced permeability and retention; MOMP: Mitochondrial outer membrane permeabilization; FBS: Fetal bovine serum; RTV: Relative tumor volume; ILS: Increased life span; MST: Median survival time; PVDF: Polyvinylidene fluoride membranes.

## Authors' contributions

CY was responsible for most of the experimental work and drafted the manuscript. ZF participated in the design, supervised this study, and was involved in revising the manuscript. HL participated in culturing cells, performing Western blot detection, and assisting in the statistical analysis. WL was involved with the animal model experiment. All authors read and approved the final manuscript.
